# Modulation of the lipidomic profile due to a lipid challenge and fitness level: a postprandial study

**DOI:** 10.1186/s12944-015-0062-x

**Published:** 2015-07-01

**Authors:** Ciara Morris, Colm M. O’Grada, Miriam F. Ryan, Michael J. Gibney, Helen M. Roche, Eileen R. Gibney, Lorraine Brennan

**Affiliations:** UCD Institute of Food and Health, University College Dublin, Belfield, Dublin 4, Ireland; UCD Conway Institute of Biomolecular and Biomedical Research, University College Dublin, Belfield, Dublin 4, Ireland

**Keywords:** Lipidomics, Oral lipid tolerance test, Fitness level

## Abstract

**Background:**

The lipid composition of plasma is known to vary due to both phenotypic factors such as age, gender and BMI as well as with various diseases including cancer and neurological disorders. However, there is little investigation into the variation in the lipidome due to exercise and/ or metabolic challenges. The objectives of this present study were (i) To identify the glycerophospholipid, sphingolipids and ceramide changes in response to an oral lipid tolerance test (OLTT) in healthy adults and (ii) To identify the effect of aerobic fitness level on lipidomic profiles.

**Methods:**

214 healthy adults aged 18–60 years were recruited as part of a metabolic challenge study. A sub-group of 40 volunteers were selected for lipidomic analysis based on their aerobic fitness level. Ceramides, glycerophospholipids and sphingomyelins were quantified in baseline fasting plasma samples as well as at 60, 120, 180, 240 and 300 min following a lipid challenge using high-throughput flow injection ESI-MS/MS.

**Results:**

Mixed model repeated measures analysis identified lipids which were significantly changing over the time course of the lipid challenge. Included in these lipids were lysophosphoethanolamines (LPE), phosphoethanolamines (PE), phosphoglycerides (PG) and ceramides (Cer). Five lipids (LPE a C18:2, LPE a C18:1, PE aa C36:2, PE aa C36:3 and N-C16:1-Cer) had a fold change > 1.5 at 120 min following the challenge and these lipids remained elevated. Furthermore, three of these lipids (LPE a C18:2, PE aa C36:2 and PE aa C36:3) were predictive of fasting and peak plasma TAG concentrations following the OLTT. Further analysis revealed that fitness level has a significant impact on the response to the OLTT: in particular significant differences between fitness groups were observed for phosphatidylcholines (PC), sphingomyelins (SM) and ceramides.

**Conclusion:**

This study identified specific lipids which were modulated by an acute lipid challenge. Furthermore, it identified a series of lipids which were modulated by fitness level. Future lipidomic studies should take into account environmental factors such as diet and fitness level during biomarker discovery work.

**Trial registration:**

Data, clinicaltrials.gov, NCT01172951

**Electronic supplementary material:**

The online version of this article (doi:10.1186/s12944-015-0062-x) contains supplementary material, which is available to authorized users.

## Background

Lipidomics characterises the composition of lipid molecular species in biological systems [1, 2]. Recent development in lipidomics research has been largely driven by the advancements in mass spectrometry (MS) and innovations in chromatographic technologies. To date lipidomic investigations have focused largely on the measurement of alterations of lipids at systems-level indicative of disease, environmental perturbations or response to diet, drugs and toxins as well as genetics [3] and thus, lipidomics is providing new opportunities to understand the function of lipids in biological systems [4]. Recent studies have demonstrated the application of lipidomics in studies investigating diabetes, cystic fibrosis, respiratory diseases and neurodegenerative diseases. These studies have clearly indicated the potential usefulness of lipidomics both to generate biomarkers of disease and to probe signalling and metabolic processes [5–9].

Lipidomic approaches have started to gain momentum in the area of nutrition and food research. Examples of use can be broadly defined into two categories: (1) application of lipidomics to further our understanding of the effects of specific diets and foods on metabolic pathways and (2) application to the study of diet related diseases [10–12]. Significant knowledge has been obtained through the application of lipidomics in obesity and type 2 diabetes research [13, 14]. Furthermore, lipidomic patterns have been linked to dietary intake revealing the potential use for identifying biomarkers of food intake [15]. Recent applications of lipidomics has revealed a new set of lipid signalling molecules that play a key role in whole body metabolism and physiology offering new targets for the treatment of type 2 diabetes and the metabolic syndrome [16].

In the field of nutrition research the use of meal challenges to characterise subjects and to identify subtle changes following interventions is emerging as an important tool in nutrition research [17–22]. Healthy humans have the ability to maintain homeostasis through a multitude of nutritionally regulated processes. Therefore time-dependant changes in response to food is of great importance and provides information about the health of an individual. Biochemical alterations can be examined using challenge tests such as a standard meal, an oral glucose tolerance test (OGTT) or an oral lipid tolerance test (OLTT). One recent study carried out by Krug and colleagues utilised both an OGTT and an OLTT in healthy men to investigate the dynamics of the human metabolome in response to different challenges with a particular focus on lipids and amino acids [18]. The study revealed that physiological challenges increase inter-individual variation even in phenotypically similar volunteers, which in turn revealed metabotypes which were not observed at baseline. While Krug’s paper identified the importance of challenging the homeostatic state, to date the literature on postprandial response of lipids after an OLTT is limited and more work is needed to progress the use of these challenge tests in nutrition research.

It is well established that exercise has a beneficial role on the lipid profile of both healthy individuals and individuals suffering from the metabolic syndrome [9, 23–25]. Early studies have shown that VO_2max_ correlates inversely with markers of the metabolic syndrome; including triacylglycerols (TAG) [26, 27], however to date there is limited data demonstrating the effects of aerobic fitness level on lipidomic profiles of healthy individuals. Furthermore, little is known on the impact of fitness level on the dynamic response to a challenge test. Therefore the objectives of the present work were to (1) determine the lipidomic response to an OLTT and (2) examine the impact of aerobic fitness on the plasma lipidomic profile.

## Results

Detailed lipidomic analysis was performed on a total of 40 subjects over the timecourse of an OLTT. The subjects had an average age of 35 ± 14 (years) and average BMI of 26.5 ± 6.3 (kg/m^2^) (Table 1). The fitness level as determined by VO_2max_ ranged from 8 ml/min/kg to 65 ml/min/kg. The low fitness group were defined as subjects with a VO_2max_ < 42 ml/min/kg and the high fitness group were defined as subjects with a VO_2max_ > 43 ml/min/kg.

**Table 1:** Demographics of study population

	Mean ± SD
Age (years)	35 ± 14
Weight (Kg)	80.76 ± 19.76
BMI (kg/m^2^)	26.5 ± 6.3
Body fat (%)	27.7 ± 13.7
VO_2MAX_ (ml/min/kg)	41.1 ± 16.2

Data are means ± SD

### Dynamic changes in the lipid profiles following an OLTT

Mixed model repeated measures analysis identified lipids which were significantly changing over time during the OLTT (Table 2). The results revealed that the greatest numbers of lipids were changing at timepoints 120 and 180 min. Sixty one lipids were significantly different between the baseline value at the 120 min timepoint. For the 180 min timepoint there were seventy six lipids significantly different from baseline measurements. Examples of the alterations during the timecourse are given in Additional file 1: Figure S1. It should also be noted that the TAG response was not significantly different between males and females (Additional file 1: Figure S2).

**Table 2:** Lipids which change significantly during an OLTT

60 min	120 min	180 min	240 min	300 min
LPE.a.C16.0	LPC.a.C16.0	LPC.a.C16.0	LPC.a.C18.1	LPC.a.C18.0
LPE.a.C20.4	LPC.a.C18.2	LPC.a.C18.0	LPC.a.C18.2	LPC.a.C18.1
N.C11.0.Cer.2H.	LPC.a.C20.4	LPC.a.C18.1	LPC.a.C20.4	LPC.a.C18.2
N.C12.1.Cer.2H.	LPE.a.C16.0	LPC.a.C18.2	LPE.a.C18.1	LPC.a.C20.4
N.C17.1.Cer	LPE.a.C18.0	LPC.a.C20.4	LPE.a.C18.2	LPC.e.C18.0
N.C19.0.OH.Cer	LPE.a.C18.1	LPC.e.C18.0	LPE.a.C20.4	LPE.a.C18.1
N.C20.0.OH.Cer	LPE.a.C18.2	LPE.a.C16.0	LPE.a.C22.6	LPE.a.C18.2
N.C21.0.OH.Cer	LPE.a.C20.4	LPE.a.C18.0	N.C12.1.Cer.2H.	LPE.a.C20.4
N.C25.0.Cer.2H.	LPE.a.C22.6	LPE.a.C18.1	N.C14.0.OH.Cer	LPE.a.C22.6
PC.aa.C32.1	N.C13.0.Cer	LPE.a.C18.2	N.C16.1.Cer	N.C13.0.Cer
PC.aa.C32.2	N.C16.1.Cer	LPE.a.C20.4	N.C21.0.OH.Cer	N.C13.1.Cer
PC.aa.C36.5	N.C20.0.Cer.2H.	LPE.a.C22.6	N.C23.0.Cer	N.C15.0.OH.Cer
PC.aa.C38.3	N.C20.0.OH.Cer	N.C13.0.Cer	N.C27.0.Cer.2H.	N.C16.1.Cer
PC.aa.C38.4	N.C20.0.OH.Cer.2H.	N.C16.1.Cer	PC.aa.C34.2	N.C21.0.Cer
PC.aa.C38.5	N.C21.0.Cer	N.C21.0.OH.Cer	PC.aa.C36.0	N.C21.0.OH.Cer
PC.aa.C38.6	N.C21.0.OH.Cer	N.C22.0.Cer	PC.aa.C36.2	N.C23.0.Cer
PC.aa.C40.4	N.C23.0.Cer	N.C23.0.Cer	PC.aa.C36.3	N.C25.0.OH.Cer
PC.aa.C40.5	N.C24.0.OH.Cer.2H.	N.C24.0.Cer	PC.aa.C36.4	N.C27.0.Cer.2H.
PC.aa.C40.6	N.C25.0.Cer	N.C25.0.Cer	PC.aa.C36.5	N.C27.0.OH.Cer
PC.aa.C40.7	N.C27.0.Cer	N.C25.0.Cer.2H.	PC.aa.C38.5	PC.aa.C34.2
PC.ae.C32.1	N.C27.0.Cer.2H.	N.C27.0.Cer.2H.	PC.aa.C38.6	PC.aa.C36.2
PC.ae.C34.3	N.C8.0.OH.Cer	PC.aa.C34.1	PC.ae.C36.2	PC.aa.C36.3
PC.ae.C36.3	PC.aa.C32.1	PC.aa.C34.2	PC.ae.C36.4	PC.aa.C36.4
PC.ae.C36.4	PC.aa.C34.1	PC.aa.C34.3	PC.ae.C38.4	PC.aa.C36.5
PC.ae.C38.2	PC.aa.C34.2	PC.aa.C36.0	PC.ae.C38.5	PC.aa.C38.3
PC.ae.C38.3	PC.aa.C34.3	PC.aa.C36.1	PC.ae.C38.6	PC.aa.C38.4
PC.ae.C38.4	PC.aa.C36.0	PC.aa.C36.2	PE.aa.C22.2	PC.aa.C38.5
PC.ae.C38.5	PC.aa.C36.1	PC.aa.C36.3	PE.aa.C36.2	PC.aa.C38.6
PC.ae.C38.6	PC.aa.C36.2	PC.aa.C36.4	PG.aa.C34.0	PC.aa.C40.6
PC.ae.C40.5	PC.aa.C36.3	PC.aa.C36.5	PG.aa.C36.0	PC.aa.C40.7
PE.aa.C22.2	PC.aa.C36.4	PC.aa.C38.3	PG.aa.C36.2	PC.ae.C34.6
PG.aa.C36.3	PC.aa.C36.5	PC.aa.C38.4	PS.aa.C36.4	PC.ae.C38.1
PS.aa.C36.4	PC.aa.C38.3	PC.aa.C38.5	PS.aa.C40.5	PC.ae.C38.2
PS.aa.C40.5	PC.aa.C38.4	PC.aa.C38.6		PC.ae.C38.3
SM.C18.1	PC.aa.C38.5	PC.aa.C40.4		PC.ae.C38.4
SM.C20.1	PC.aa.C38.6	PC.aa.C40.5		PC.ae.C38.5
SM.C20.2	PC.aa.C40.4	PC.aa.C40.6		PC.ae.C38.6
SM.C21.0	PC.aa.C40.5	PC.aa.C40.7		PE.aa.C22.2
SM.C21.1	PC.aa.C40.6	PC.ae.C32.0		PE.aa.C36.2
SM.C22.0	PC.aa.C40.7	PC.ae.C32.1		PE.aa.C36.3
SM.C22.1	PC.ae.C32.1	PC.ae.C34.1		PG.aa.C34.0
SM.C23.0	PC.ae.C36.4	PC.ae.C34.2		PG.aa.C36.1
SM.C23.1	PC.ae.C36.5	PC.ae.C36.2		PG.aa.C36.2
	PC.ae.C38.3	PC.ae.C36.3		PG.ae.C34.1
	PC.ae.C38.4	PC.ae.C36.4		PS.aa.C36.4
	PC.ae.C38.5	PC.ae.C36.5		PS.aa.C40.5
	PC.ae.C38.6	PC.ae.C38.2		PS.aa.C42.1
	PC.ae.C40.5	PC.ae.C38.3		SM.C3.0
	PE.aa.C22.2	PC.ae.C38.4		
	PE.aa.C36.2	PC.ae.C38.5		
	PG.aa.C32.0	PC.ae.C38.6		
	PG.aa.C33.6	PC.ae.C40.5		
	PG.aa.C34.1	PE.aa.C22.2		
	PG.aa.C36.1	PE.aa.C36.2		
	PG.aa.C36.2	PE.aa.C36.3		
	PS.aa.C36.4	PG.aa.C34.1		
	PS.aa.C40.5	PG.aa.C34.2		
	SM.C20.2	PG.aa.C36.1		
	SM.C21.1	PG.aa.C36.2		
	SM.C23.0	PG.ae.C36.1		
	SM.C3.0	PS.aa.C36.4		
		PS.aa.C40.5		
		SM.C16.0		
		SM.C16.1		
		SM.C18.0		
		SM.C18.1		
		SM.C20.0		
		SM.C20.1		
		SM.C20.2		
		SM.C21.0		
		SM.C22.0		
		SM.C22.1		
		SM.C23.0		
		SM.C23.1		
		SM.C24.2		
		SM.C3.0		

Mixed model regression analysis was performed using the lme4 package in the R programming language for statistical computing, R version 2.15.0 and lme4 version 0.999999-0. Each post prandial time point was compared to base line value *p* < 0.05 was considered significantly different

**Fig. 1:**
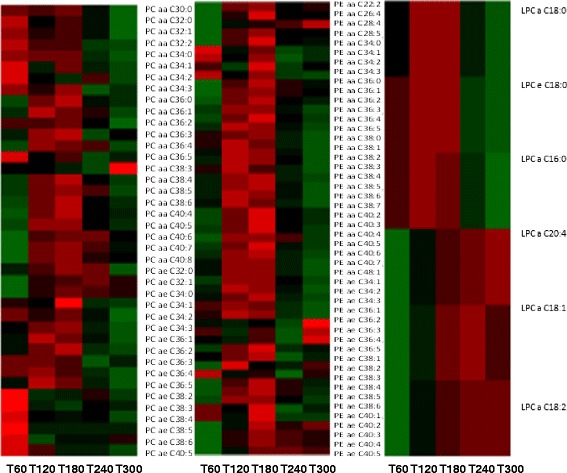
Heat maps showing the fold change of PCs, PEs and LPCs which change significantly from baseline value at each time point during the OLTT. Heat map visualisation of PCs, PEs and LPCs which change significantly from baseline value at each time point during the OLTT. Fold changes from baseline were calculated and are represented in the heat map; Red represents a positive fold change, green represents a negative fold change

Fold change analysis revealed that 12 lipids had a fold change > 1.5 compared to fasting values. Five lipids namely LPE a C18:2, LPE a C18:1, PE aa C36:2, PE aa C36:3 and N-C16:1-Cer, all had a fold change of > 1.5 at 120 min and this fold change remained for the timecourse of the OLTT. N-C10:0(OH)-Cer(2H) and N-C26:0-Cer(2H) had a fold change >1.5 at 60 min, PE aa C36:1, PG aa C34:1 and N-C24:0(OH)-Cer(2H) had a fold change of >1.5 at 180 min and N-C25:0(OH)-Cer fold change >1.5 at 300 min. Finally PG aa C36:2 had a fold change of >1.5 at 120 and 180 min. Visualisation of the changes was achieved using heat maps (Fig. 1). The heat maps from the PEs and the PCs concur that the greatest fold change differences were at 120 and 180 min. Interestingly the saturated LPCs had increased fold differences at 60 min while the unsaturated LPC had decreased fold changes compared to baseline at 60 min. These fold changes where inverted at 300 min with the saturated LPCs having a negative fold change at 300 min while the unsaturated LPCs had a positive fold changes at the final time point measured.

Linear regression analysis of the baseline values of five lipids with a fold change greater than 1.5 at 120 min and for the remainder of the OLTT revealed that three of the lipids, LPE a C18:2, PE aa C36:2 and PE aa C36:3, were predictive of TAG concentrations both at fasting and at peak during the OLTT (Additional file 1: Tables S1 and S2).

### Impact of fitness level on the lipidomic profile

Biochemical, metabolic and inflammatory parameters for both groups are shown in Table 3. Age, BMI, weight, percentage body fat, fitness level and HOMA-IR were significantly different between the low and high fitness group. There were no significant differences observed in glucose concentration, insulin, or cholesterol. Analysis of the impact of fitness level on the fasting lipidomic profile revealed 9 lipids that were significantly lower in the low fitness compared to the high fitness group (Table 4). However, analysis of the response to the OLTT revealed significant interactions between time and fitness group for a greater number of lipids (Table 5). Interestingly, the timecourse analysis revealed that time points 180 and 300 min had the greatest number of group x time interactions. The lipid classes which had significant group x time interaction at180 and 300 min were; PCs, SM and ceramides. Further analyses revealed that the changes in response to the lipid challenge were more pronounced in the high fitness group.

**Table 3:** Biochemical, metabolic and inflammatory parameters for the low and high fitness groups

	Low fitness	High fitness	*P* Value
Gender M/F	11/11	9/9	ns
Age (Years)	39 ± 14	29 ± 11	0.017
Weight (Kg)	87.96 ± 22.69	71.96 ± 10.54	0.009
BMI (kg/m^2^)	28.95 ± 7.23	23.54 ± 3.16	0.005
Percentage body fat (%)	34.31 ± 14.12	19.96 ± 8.05	0.001
VO_2MAX_ (ml/min/kg)	28.5 ± 7.4	54.4 ± 7.0	<0.001
C Peptide (ng/ml)	4.58 ± 3.3	2.31 ± 1.90	0.114
Insulin (μIU/ml)	14.74 ± 9.36	7.86 ± 5.23	0.072
Glucose (mmol/l)	5.62 ± 1.04	5.54 ± 0.46	0.837
Cholesterol (mmol/l)	4.97 ± 0.99	4.61 ± 1.09	0.285
HDL Cholesterol (mmol/l)	1.40 ± 0.43	1.42 ± 0.37	0.893
Triglycerides (mmol/l)	1.31 ± 0.60	1.01 ± 0.36	0.067
HOMA-IR	3.94 ± 2.51	1.80 ± 1.33	0.040
TNFα (pg/ml)	5.55 ± 3.31	6.11 ± 5.36	0.693
IL6 (pg/ml)	2.29 ± 2.55	1.10 ± 0.40	0.094

Data are means ± SD. *P* values are based on ANOVA between fitness groups

**Table 4:** List of baseline differences in lipids between low and high fitness groups

	Low fitness	High fitness	*P* Value
PE ae C34:3	0.136 ± 0.039	0.169 ± 0.041	0.017
PE ae C36:3	0.381 ± 0.144	0.485 ± 0.138	0.046
PC ae C34:2	6.027 ± 2.344	8.783 ± 3.605	0.008
PC ae C34:3	4.035 ± 1.836	6.949 ± 3.156	0.004
PC ae C36:3	3.988 ± 2.108	6.159 ± 2.163	0.027
PC ae C36:4	11.600 ± 2.350	14.015 ± 3.279	0.043
PC ae C36:5	9.067 ± 2.886	11.535 ± 3.542	0.044
PC ae C38:2	0.727 ± 0.416	1.305 ± 0.509	0.025
N-C10:0-Cer	0.031 ± 0.012	0.042 ± 0.016	0.035

Data are means ± SD (μM). *P* values are based on multivariate GLM of between group comparisons adjusted for age and BMI and corrected for multiple comparisons

*PE* phosphoethanolamines, *PC* phosphatidylcholine, *Cer* ceramide

**Table 5:** Lipids displaying a differential response according to fitness level during an OLTT

60 min	120 min	180 min	240 min	300 min
N.C10.1.Cer	N.C11.0.Cer	LPC.a.C16.0	LPC.a.C16.0	LPC.a.C16.0
N.C19.1.Cer	N.C11.0.OH.Cer.2H	LPC.a.C18.0	LPC.a.C18.1	LPC.a.C18.0
N.C21.0.OH.Cer	N.C12.0.OH.Cer	LPC.a.C18.1	LPC.a.C20.4	LPC.a.C18.1
PC.aa.C32.2	N.C14.0.Cer	LPC.a.C20.4	LPE.a.C20.4	LPC.a.C18.2
PG.aa.C36.3	N.C15.0.OH.Cer	LPC.e.C18.0	LPE.a.C22.6	LPC.a.C20.4
SM.C21.1	N.C21.0.OH.Cer	LPE.a.C16.0	N.C10.0.Cer	LPC.e.C18.0
	N.C21.1.Cer	LPE.a.C18.0	N.C14.0.Cer	LPE.a.C18.0
	N.C25.0.Cer.2H	LPE.a.C20.4	N.C14.0.OH.Cer	LPE.a.C20.4
	N.C27.0.OH.Cer.2H	LPE.a.C22.6	N.C14.1.Cer	LPE.a.C22.6
	N.C8.0.OH.Cer	N.C10.0.Cer	N.C17.1.Cer.2H	N.C10.0.Cer
	PC.ae.C34.6	N.C12.0.Cer	N.C19.1.Cer	N.C12.0.Cer
	PG.aa.C32.0	N.C14.0.Cer	N.C21.0.OH.Cer	N.C14.0.Cer
		N.C16.1.Cer	PC.ae.C34.3	N.C14.0.Cer.2H.
		N.C21.0.OH.Cer.2H	PG.aa.C34.0	N.C14.0.OH.Cer
		N.C25.0.Cer.2H.	PG.aa.C36.3	N.C14.1.Cer
		N.C27.0.OH.Cer.2H		N.C15.0.OH.Cer
		PC.aa.C32.1		N.C21.0.OH.Cer
		PC.aa.C32.2		N.C23.0.Cer
		PC.aa.C34.1		N.C23.0.OH.Cer.2H.
		PC.aa.C34.2		N.C9.0.OH.Cer.2H.
		PC.aa.C34.3		PC.aa.C32.1
		PC.aa.C36.1		PC.aa.C34.2
		PC.aa.C36.2		PC.aa.C34.3
		PC.aa.C36.3		PC.aa.C36.2
		PC.aa.C36.4		PC.aa.C36.3
		PC.aa.C36.5		PC.aa.C36.4
		PC.aa.C38.3		PC.aa.C36.5
		PC.aa.C38.4		PC.aa.C38.3
		PC.aa.C38.5		PC.aa.C38.6
		PC.aa.C38.6		PC.aa.C40.6
		PC.aa.C40.4		PC.ae.C34.2
		PC.aa.C40.5		PC.ae.C34.3
		PC.aa.C40.6		PC.ae.C34.6
		PC.ae.C34.1		PC.ae.C36.3
		PC.ae.C34.2		PC.ae.C36.4
		PC.ae.C34.3		PC.ae.C38.1
		PC.ae.C36.3		PC.ae.C38.2
		PC.ae.C36.4		PC.ae.C38.5
		PC.ae.C36.5		PC.ae.C38.6
		PC.ae.C38.2		PE.aa.C22.2
		PC.ae.C38.4		PS.aa.C42.1
		PC.ae.C38.5		SM.C16.1
		PC.ae.C40.5		SM.C18.1
		SM.C14.0		SM.C19.0
		SM.C16.1		SM.C21.0
		SM.C18.0		SM.C23.1
		SM.C18.1		
		SM.C19.0		
		SM.C20.0		
		SM.C21.0		
		SM.C22.0		
		SM.C23.1		

Variables were analysed using mixed model analysis. Timepoint differences *p* < 0.05 were considered significantly different

## Discussion

The present study revealed a significant impact of exercise on the lipidomic profile and described the postprandial lipidomic profile. Moreover, detailed analysis of the dynamic response to the lipid challenge supports the hypothesis that examination of metabolites/proteins in the postprandial state reveals more information compared to analysis in the fasting state [28].

Throughout the day, people are primarily in a postprandial state; thus fasting lipid values may not always reflect the relative risk of cardiovascular related diseases. There is therefore a need to move to analysis of lipids following challenge tests such as the OLTT. The present study adds to our knowledge of the modulation of lipid profiles following dietary challenges. Fold change analysis revealed that the largest changes compared to baseline during an OLTT were seen in PEs, LPEs, PGs, and ceramides. In particular LPE a C18:2, LPE a C18:1, PE aa C36:2, PE aa C36:3 and N-C16:1-Cer, all had a fold change of > 1.5 at 120 min and this fold change remained >1.5 for the remainder of the OLTT. The PE family of lipids represent about 25 % of mammalian phospholipids and in recent years their importance in a number of cellular processes has emerged: PE is now recognised as being important for mitochondrial function, mitochondrial morphology and autophagy [29–31]. The importance of the significant increase in these lipids during an OLTT remains to be resolved. Further analysis of the 5 lipids with a fold change > 1.5 revealed that baseline values of LPE a C18:2, PE aa C36:2, PE aa C36:3 were predictive of fasting and peak TAG concentration. There is a large body of evidence that demonstrates that postprandial hypertriglyceridemia is an independent risk for cardio vascular disease [32–34]. Relationship of the specific LPE and PEs with the TAG concentrations is interesting in light of the fact that plasma concentrations of LPE a C18:2 were found to be elevated in subjects with diabetes [35]. Further investigation into the role of LPE and PE in cardiovascular diseases is warranted.

The present analysis revealed that the dynamic changes in LPCs depended on the saturation level of the LPC. Unsaturated LPCs had decreased fold changes compared to baseline at 60 min while saturated LPCs had increased fold changes compared to baseline at 60 min. These fold changes were inverted at 300 min with the saturated LPCs having a negative fold change at 300 min while the unsaturated LPCs had a positive fold changes at the final time point measured. The dynamic changes in the LPCs are noteworthy considering the reports that LPC levels are reduced in individuals with impaired glucose tolerance [36] and that LPC C16:0 is reduced in insulin resistant subjects with non-alcoholic fatty liver disease [37]. Additionally, in a high fat feeding animal study significant and rapid changes in LPC levels were observed leading the authors to suggest that diet and adiposity may be drivers of the LPC levels [38]. Interestingly the timecourse analysis in the animal study revealed a differential pattern of change for the saturated LPCs agreeing with the results found in humans in the present study. Furthermore, a recent study has demonstrated that lysophosphatidic acid exerts a deleterious effect on glucose disposal in high fat diet induced obesity [39]. Further work is needed to clarify the importance of diet for the fasting levels of LPCs.

Analysis of the effect of fitness level on the fasting lipidomic profile revealed that long chain phosphatidylcholines (PCs) were significantly different between the low and high fitness groups. Six phosphatidylcholines were significantly lower in the lower fitness group. This is in agreement with a recent study [40] in which Bye and colleagues’ concluded low VO_2max_ subjects had decreased serum concentrations of phosphatidylcholines compared to those with high VO_2max_. In this work, Bye suggested that due to the lower concentrations of phosphatidylcholines, healthy individuals with a low aerobic fitness may have a high Phospholipase D activity, which may link low aerobic fitness to the future development of CVD. Our study revealed that exploration of dynamic changes following a high fat challenge revealed more significant differences according to fitness level compared to analysis at baseline or fasting state. This concept has emerged in the literature and has been demonstrated in the context of dietary interventions [28].

Analysis of the dynamic changes revealed the greatest changes in LPEs, PEs and Ceramides according to fitness level. Previous research has shown that exercise has a beneficial role on lipid profile after a dietary challenge [41–45], whilst detraining in athletes rapidly increases postprandial TAG response to the level of untrained subjects [41, 42]. The present data revealed that the change in the lipids following the lipid challenge was more pronounced for the higher fitness group. This concurs with recently published data where an intervention which modified dietary fat revealed that the extent to which dietary fatty acids penetrate cellular lipids depended on fitness level [46]. The authors suggest that the strong interaction between diet and fitness level may be due to adaptations in muscle lipid metabolism [46]. The differential response to the dietary challenge according to fitness level is particularly important considering the controversy surrounding the role of lipid species in the development of muscle insulin resistance: some studies report that increased concentrations of diacylglycerols are associated with insulin resistance while others have demonstrated that concentrations are higher in trained athletes [47, 48]. The present work supports a hypothesis that the alterations in lipids in response to dietary challenges may be more important than fasting concentrations and the ability of high fitness subjects to change lipid pools in the postprandial state may confer beneficial effects.

## Conclusion

In conclusion this study has identified the key lipids which change over the course of an OLTT and identified a pattern of LPE and PE lipids that are significantly related to the TAG response. Significant changes due to fitness level were observed and our data supports the view that examination of profiles in the challenge state reveals more information compared to analysis in the homeostatic state.

## Methods

This study is part of the Metabolic Challenge Study (MECHE) as previously described [49]. Ethical approval was obtained from the Research Ethics Committees in University College Dublin (UCD) (LS-08-43-Gibney-Ryan).

Two hundred and fourteen subjects aged 18–60 years were recruited following a screening which included evaluation of the following parameters: fasting glucose, TAG, haemoglobin, and HDL- and LDL- cholesterol concentrations. Individuals with a BMI <18.5 kg/m^2^, low hemoglobin (<12 g/dL), elevated fasting glucose (FPG ≥7 mmol/l), cholesterol (>7.5 mmol/l), TAG (>3.8 mmol/l) or exhibiting raised liver or kidney enzymes, any of which warranted pharmaceutical treatment, were excluded as previously reported [49]. Subjects were informed about the experimental procedures and purpose of the study prior to giving written consent. Subjects were randomised to one of three groups; 75 subjects were randomised to receive an OGTT and an OLTT on two separate clinical visits. 69 subjects were randomised to two OGTTs on two separate clinical visits. Finally 70 subjects were randomised two OLTTs on two separate clinical visits. For the present study a sub-cohort of 40 subjects (20 male, 20 female) were the upper and lower quartiles of the estimated VO_2max_ levels for subjects who underwent at least 1 OLTT.

Following a 12 h overnight fast, second void urine and blood samples were collected. The urine was immediately centrifuged at 1800 g for 10 min at 4 °C, and 1 ml aliquots were stored at −80 °C. All individuals underwent a 75 g OGTT according to the recommendations of the World Health Organization (WHO)/International Diabetes Federation IDF. Venous blood samples were obtained directly before (=0 min) and at 10, 20, 30, 60, 90, and 120 min during the OGTT. The OLTT consisted of 100 mL Calogen (Nutricia, Ireland) combined with 50 mL Liquid Duocal (SHS Nutrition, Netherlands) for a total of 150 mL with a fat content of 54 g fat (7 g SFA, 31 g MUFA, 16 g PUFA) and 11 g carbohydrate [49].

Venous blood samples were obtained directly before (=0 min) and at 60, 120, 180, 240, and 300 min during the OLTT.

Serum and plasma samples were collected using serum tubes containing a clot activator coating, EDTA-coated evacuated tubes and tubes containing lithium heparin. The serum samples were allowed to clot for 30 min at room temperature. EDTA and lithium heparin tubes were placed directly on ice. All blood samples were centrifuged at 1800 *g* for 15 min at 4 °C and 500 μl aliquots were stored at −80 °C until subsequent analysis.

### Body composition and fitness tests

Body composition and cardiovascular fitness assessment were performed as described previously [50]. Height was measured using a wall-mounted stadiometer. Weight was measured on a calibrated beam balance platform scale. Percentage body fat was measured via an air-displacement plethysmograph, which has been shown to be an accurate method for assessing body composition in adults [51]. The system used in this study was the BOD POD body composition system (Model 200OA; Life Measurements Instruments, Concord, CA, USA). Percentage body fat measurements were measured in the fasting state. The VO_2max_ test was carried out after the volunteer received a breakfast consisting of 1 × 250 ml orange Fruice drink, 1 × strawberry Kellogg’s® Nutri grain® Cereal bar, 1 × medium banana and 1 × 125 g Dale Farm Spelga low fat bio yoghurt [50]. To individualise the increment of exercise intensity during the VO_2max_ test, the workload of each step was calculated from the theoretical VO_2max_ using the Jones equation [52]. Consequently subjects underwent a test with the same relative incremental workload. The test consisted of a three minute warm up at baseline followed by four minute steady state workloads at 15, 35, 55, and 75 %. Recovery to baseline (VO_2_ and heart rate returned to baseline value and an R value under 1 was achieved) was recorded after each increment. The subjects performed the test on an electronically braked cycle ergometer (Ergoline Bosh 500). Heart rate was monitored continuously throughout the test and metabolic and ventilatory responses were assessed using a computer based breath to breath exercise analysing system (Quark b2, Cosmed, Rome, Italy).

### Lipidomic analysis

Lipidomics analysis was carried out by BIOCRATES Life Sciences AG (Biocrates Life sciences AG, Innsbruck, Austria) on lithium heparin plasma samples. The biologically most abundant members of (lyso-) glycerophospholipids, i.e. (lyso-) glycerolphosphocholines, −ethanolamines, −serines, −glycerols, as well as sphingolipids, i.e. sphingomyelins, ceramides, dihydroceramides, and 2-hydroxyacyl ceramides, were quantitatively analysed by a high throughput flow injection ESI-MS/MS method as previously described [15, 53]. The quantitative data analysis was performed with Biocrates in-house software MetIDQ™ enabling isotopic correction and basic statistical analysis. To deal with data at or below the limit of detection only lipids which had ≥ 70 % of subjects above the limit of detection were included in this analysis [54]. Therefore from the 325 lipids that were measured, 240 lipids were included for data analysis. The median intra-day CV was < 8 % taking into account all measured lipids. The median inter-day CV was 16 % taking into account all lipids.

### Biochemical and immune parameters

Clinical chemistry analysis was performed using an RxDaytona™ chemical analyser autoanalyser (Randox Laboratories, Crumlin, UK) and Randox reagents as previously described [49]. Details of the analytes and methods are as follows: total cholesterol (cholesterol oxidase), HDL-cholesterol (direct clearance), glucose (glucose oxidase), triacylglycerol (lipase/glycerol kinase colorimetric), C reactive protein (immunoturbidimetric) and NEFA (lipase/glycerol kinase colorimetric).

The Evidence Investigator™ (Randox Laboratories, Crumlin, Northern Ireland) metabolic array I kit was used for the simultaneous measurement of C-peptide, Insulin, Resistin, and Tumor Necrosis Factor-a (TNFa). Standard quality control procedures were followed on both analysers to ensure the integrity of the data.

HOMA-IR score was calculated using the formula; (Fasting insulin μU/mL x fasting glucose mmol/L)/ 22.5 and Quicki score was calculated using the formula; 1 / (log (fasting insulin μU/mL) + log(fasting glucose mg/dL).

### Statistical analysis

Descriptive statistics, ANOVA and general linear model analyses were performed using PASW version 18.0 for windows. *P* values were corrected for multiple comparisons using a Bonferroni correction where appropriate. Data are presented as mean ± standard deviation (SD). Mixed model regression analysis was done using the lme4 package in the R programming language for statistical computing, R version 2.15.0 and lme4 version 0.999999-0, both available under the GNU public license (http://www.cran.r-project.org.) Lme4 is an R package for fitting and analysing linear, non linear and generalised linear mixed models. Individual lipid concentrations were visualised using the beanplots, implemented in ‘beanplot’ R package. Heatmaps were produced in R for visualisation of fold changes in lipid concentrations.

## Additional file

Additional file 1:
**Table S1.** Lipids predictive of fasting triacylglycerols. **Table S2.** Lipids predictive of peak triacylglycerols during an OLTT. **Figure S1.** Time response of representative lipids and the TAG response over the time course of the OLTT. **Figure S2.** Time response of TAG by gender.

## Abbreviations

HOMA: Homeostatic model assessment
NEFA: Non-esterified fatty acids
TAG: Triglyceride
