# Supplementary material for: Modulation of the lipidomic profile due to a lipid challenge and fitness level: a postprandial study
Source: Lipids Health Dis. 2015 Jul 1;14:65. doi: 10.1186/s12944-015-0062-x (PMC4489019; doi:10.1186/s12944-015-0062-x)
Supplement: Additional file 1: — Table S1. Lipids predictive of fasting triacylglycerols. Table S2. Lipids predictive of peak triacylglycerols during an OLTT. Figure S1. Time response of representative lipids and the TAG response over the time course of the OLTT. Figure S2. Time response of TAG by gender. [file 12944_2015_62_MOESM1_ESM.docx]

**Additional file 1**

**Table S1. Lipids predictive of fasting triacylglycerols**

|  | **Unstandardized Coefficients** | | **Standardized Coefficients** | **t** | **P Value** |
| --- | --- | --- | --- | --- | --- |
|  | **B** | **Std. Error** | **Beta** |  |  |
|  |  |  |  |  |  |
| **LPE a C18:2** | -1.182 | 0.494 | -0.351 | -2.391 | 0.022 |
| **PE aa C36:2** | 0.401 | 0.099 | 1.219 | 4.069 | <0.001 |
| **PE aa C36:3** | -0.684 | 0.184 | -1.105 | -3.727 | 0.001 |

**Table S2. Lipids predictive of peak triacylglycerols during an OLTT**

|  | **Unstandardized Coefficients** | | **Standardized Coefficients** | **t** | **P value** |
| --- | --- | --- | --- | --- | --- |
|  | **B** | **Std. Error** | **Beta** |  |  |
|  |  |  |  |  |  |
| **LPE a C18:2** | -2.657 | 1.003 | -0.382 | -2.649 | 0.012 |
| **PE aa C36:2** | 0.842 | 0.200 | 1.237 | 4.210 | <0.001 |
| **PE aa C36:3** | -1.425 | 0.372 | -1.113 | -3.827 | <0.001 |

**Figure S1**. Time response of representative lipids and the TAG response over the time course of the OLTT.

**Figure S2**. Time response of representative TAG by gender.
